# Supplementary figures and images for: Evolution and multiple roles of the Pancrustacea specific transcription factor zelda in insects
Source: PLoS Genet. 2017 Jul 3;13(7):e1006868. doi: 10.1371/journal.pgen.1006868 (PMC5515446; doi:10.1371/journal.pgen.1006868)

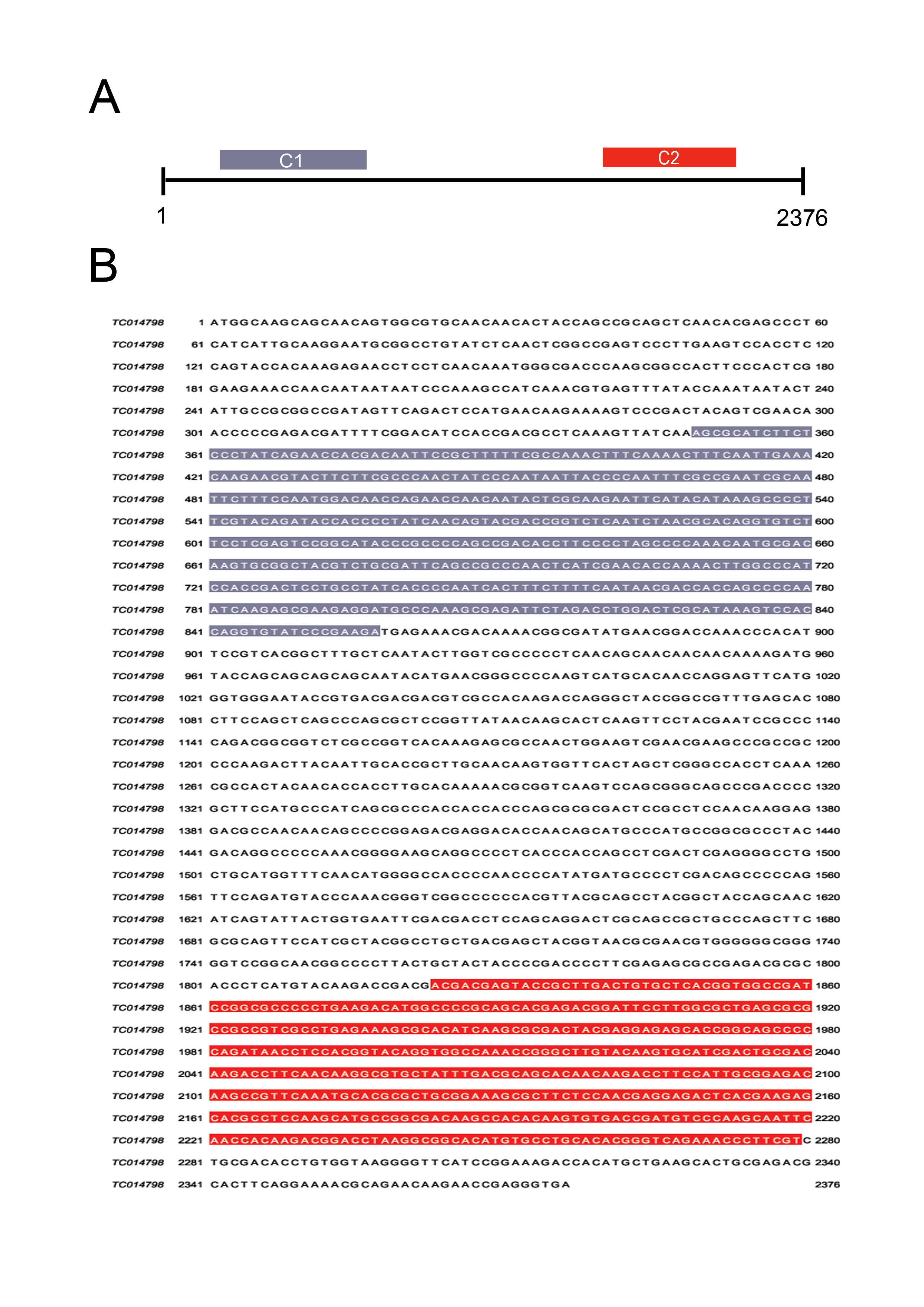

Supplement: S1 Fig — Tc-zld gene corresponds to the Beetlebase ID: TC014798. The following primer pairs were used: C1-Forward ggccgcggAGCGCATCTTCTCCCTATCA and C1-Reverse cccggggcGCCGTTTTGTCGTTTCTCAT. This primer pair amplifies leads to the amplification of a fragment of 528 bp (349–857). A second primer pair C2-Forward ggccgcggACGACGAGTACCGCTTGACT and C2-Reverse—cccggggcCTTACCACAGGTGTCGCAGA was also used and lead to similar results. This primer pair amplifies a fragment of 476 bp (1823–2279) covering the four zinc-finger domains. The lowercase letters contain the primer sequence used as a template for a second PCR using universal primers which adds a T7 promoter at both sides of the PCR template as previously described [81]. (TIF) [file pgen.1006868.s001.tif]
